# Beyond Conventional Monitoring: A Semantic Segmentation Approach to Quantifying Traffic-Induced Dust on Unsealed Roads

**DOI:** 10.3390/s24020510

**Published:** 2024-01-14

**Authors:** Asanka de Silva, Rajitha Ranasinghe, Arooran Sounthararajah, Hamed Haghighi, Jayantha Kodikara

**Affiliations:** 1 ARC Industrial Transformation Research Hub (ITRH)—SPARC Hub, Department of Civil Engineering, Monash University, Clayton Campus, Clayton, VIC 3800, Australia; senarathna.desilva@monash.edu (A.d.S.); rajitha.ranasinghe@monash.edu (R.R.); arooran.sounthararajah@monash.edu (A.S.); 2 Product Development Hub, Road Science, Downer EDI Works Pty Ltd., Somerton, VIC 3061, Australia; hamed.haghighi@downergroup.com

**Keywords:** dust, dust monitoring, dust quantification, deep learning, machine learning, road dust, semantic segmentation, traffic-induced dust, unsealed roads

## Abstract

Road dust is a mixture of fine and coarse particles released into the air due to an external force, such as tire–ground friction or wind, which is harmful to human health when inhaled. Continuous dust emission from the road surfaces is detrimental to the road itself and the road users. Due to this, multiple dust monitoring and control techniques are currently adopted in the world. The current dust monitoring methods require expensive equipment and expertise. This study introduces a novel pragmatic and robust approach to quantifying traffic-induced road dust using a deep learning method called semantic segmentation. Based on the authors’ previous works, the best-performing semantic segmentation machine learning models were selected and used to identify dust in an image pixel-wise. The total number of dust pixels was then correlated with real-world dust measurements obtained from a research-grade dust monitor. Our method shows that semantic segmentation can be adopted to quantify traffic-induced dust reasonably. Over 90% of the predictions from both correlations fall in true positive quadrant, indicating that when dust concentrations are below the threshold, the segmentation can accurately predict them. The results were validated and extended for real-time application. Our code implementation is publicly available.

## 1. Introduction

Dust emissions from unsealed roads occur due to the application of an external force or combined action of such forces. These external forces can be vehicle tire–ground friction and shear due to vehicle movement and wind. When road dust is airborne, it is sometimes referred to as fugitive dust or particulate matter (PM). We use dust and particulate matter interchangeably in this article. PM_x_ refers to particulate matter with a diameter of *x* μ m or less [1]. Due to the different sizes of PM causing different types of health issues, this study focuses on five particle sizes, PM_30_, PM_10_, PM_4_, PM_2.5_, and PM_1_. Notably, the inhalable (PM_10_) and respirable (PM_2.5_) PM can induce adverse health effects [2], thus are often focused on more in dust-related studies [3,4,5].

Dust emissions can occur due to aerodynamic entrainment, saltation bombardment, and aggregates disintegration, albeit the aerodynamic entrainment is often assumed to be negligible [6]. Many questions relating to dust emissions, particularly due to traffic, remain unanswered as research groups primarily focus on wind-induced erosion events that cause dust emissions [7,8,9]. The chaotic nature of dust emissions makes it difficult to use the empirical relationships [10] derived from field experiments to explain the physics involved in the process. This paper approaches the problem by considering recent advancements in machine learning (ML) to find a more pragmatic solution [11,12]. There are other unconventional attempts to quantify dust or gravel loss using smartphones [13] and machine learning algorithms [14], but none have conducted extensive field experiments and correlated that with outputs from image processing. When dust particles are airborne, they form a cloud and depending on the intensity, it can be captured in an image or a video, allowing us to perform semantic segmentation. The segmented images can then be post-processed to calculate the number of dust pixels in them. In this study, we investigate if the calculated dust pixels can be correlated with the actual field dust measurements. This is further elaborated in Section 2. The devised experimental procedures are explained in Section 3.

### 1.1. Machine Learning Approach

In machine learning, segmentation refers to the process of dividing an image into segments and automatically identifying pre-learned objects or characteristics. Semantic segmentation involves splitting the image down to its smallest component, the pixel, and assigning a label to each pixel such that pixels with the same label share certain characteristics. This process effectively enables the model to understand and interpret every part of the image with detail.

The present study study introduces a novel approach to semantic segmentation, specifically tailored for the identification and analysis of dust clouds on unsealed road surfaces. Our methodology encompasses the development and training of machine learning models, which are optimized for semantic segmentation tasks. The process is outlined as follows:

1. Data collection: The initial phase involved acquiring high-resolution images of traffic-induced dust clouds from unsealed roads, exhibiting different levels of dust accumulation. These images constitute the foundational dataset required for training the semantic segmentation model. The dataset produced is called URDE and has been published [15].
2. Annotation of dust particles: A critical step in data preparation was the manual annotation of dust particles in the collected images. Skilled annotators marked areas of dust accumulation, creating grayscale masks. These masks depicted the intensity of dust accumulation at the pixel level, thus providing a detailed representation of dust distribution within the cloud.
3. Production of binary masks: Since the semantic segmentation model necessitates binary masks, we utilized Otsu’s thresholding method to transform the detailed grayscale masks into binary format. Otsu’s method [16], an adaptive thresholding technique, determines the optimal threshold value for binarization, resulting in binary masks that categorize each pixel as either ‘dust’ or ‘non-dust’.
4. Model training: The binary masks were then used as ground truths in training our semantic segmentation models. We employed various deep learning frameworks, particularly leveraging Convolutional Neural Networks (CNNs), which are highly effective in image analysis and segmentation tasks. The models were trained to discern dust-laden areas from dust-free zones, thus learning to identify and segment dust particles within the environment.

Semantic segmentation has been used to solve road-related problems such as identifying road boundaries for autonomous vehicles [17], preventing pedestrian collision [18], and detecting road surface damages [19]. Our case of road dust differs from the cases above in that the boundary of the object of interest, i.e., dust cloud, may not be as clear as a rigid object. Dust clouds have fuzzy boundaries that are difficult to determine, and current knowledge distillation methods fail to transfer boundary information, resulting in poor boundary identification explicitly. Image segmentation could accurately determine the exact boundary of the objects in the image. In recent years, knowledge distillation for semantic segmentation has been extensively studied to obtain satisfactory performance while reducing computational costs and developing advanced ML models. Ranging from simple encoder–decoder structures to more densely connected convolutional networks [20], the machine intelligence has pushed state-of-the-art forward to advanced ML models such as MobileNetV3 [21], DeeplabV3 with resnet backbone, and fully convolutional network (FCN) with resnet backbone [22,23,24]. As our primary goal is to quantify dust in real-time using semantic segmentation, emphasis was put on video sequences and static images. However, we were unable to use these image-based segmentation methods directly on a video sequence to independently segment each frame as the segmentation results will be inconsistent for different frames due to the lack of temporal coherence limitations. For video segmentation, spatial and temporal dimensions should be considered [25]. This is further elaborated in Section 4.

### 1.2. Objectives

The objectives of this study are to:

1. Employ advanced semantic segmentation algorithms, specifically DeepLabV3, FCN with ResNet101 backbone, and Lraspp_MobileNetV3_large, to accurately identify and quantify traffic-induced dust clouds in images of unsealed roads.
2. Establish correlations between the dust pixel features extracted from the semantic segmentation process and in situ dust measurements, thereby estimating the concentration of dust.
3. Validate the effectiveness of the semantic segmentation approach in dust quantification through comprehensive field experiments, ensuring the reliability and practical applicability of the methodology.
4. Demonstrate the potential of the proposed method in real-world scenarios, particularly in monitoring and managing dust levels on unsealed roads, thereby contributing to environmental studies.

### 1.3. Structure of the Paper

Section 2 defines the scope of a problem and formulates the question, “Is the relationship between the pixel representation of the dust cloud in an image and the actual dust cloud?”, and designs the experiments needed to be conducted to gather data to address said question. Section 2 also presents the procedure we used to incorporate dust segmentation for video sequences where dust is accurately segmented in each frame and combined to form a consistent video sequence. Section 3 presents the field experimental setup and equipment used. Section 4 develops the correlation between the actual dust measurements and the dust pixel percentage calculated from semantic segmentation. Results and validation are presented in Section 5, where the effectiveness of the proposed method is demonstrated by validating the dust segmentation for independent video sequences and images, illustrating how the findings of this study can be implemented in the real world. Concluding remarks are presented in Section 6.

## 2. Problem Formulation

**Figure 1:**
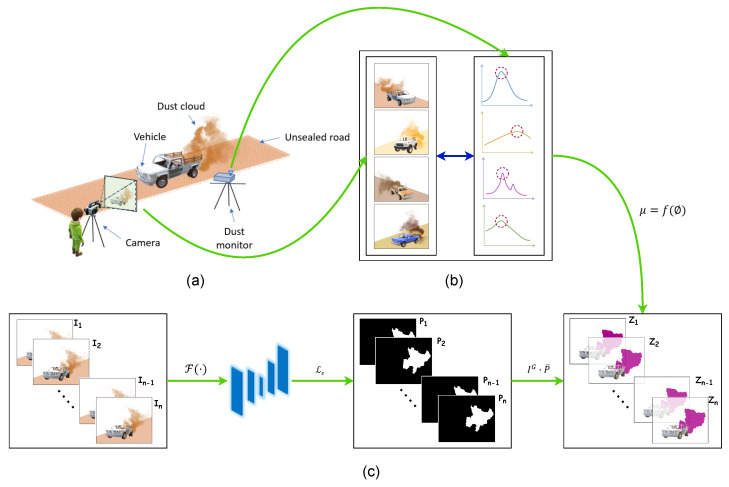
Problem visualization. (**a**) Approach to field data collection, (**b**) correlating visual features extracted from images with in situ dust measurements from the dust monitor, (**c**) inference pipeline. I is the image frame in a video sequence, F is the trained ML model, $L_{s}$ is the loss function, P is the prediction from ML model, Z is the Hadamard product between the green channel of I ( $I^{G}$ ) and the inversion of the prediction ( $\bar{P}$ ).

Suppose that a vehicle has produced a visible dust cloud. A camera was used to capture the dust emission from beginning to end, and a dust monitor was used to measure the aerosol. The process is illustrated in Figure 1. The problem is formulated based on the fact that the pictures do not and cannot represent a reality independent of the observer who sees them; thus, the following assumptions were made. The following boundary ranges were intentionally selected over discrete limits to introduce data variability.

- The dust cloud captured in the 2D image and the frame average of accumulated probability densities of dust (ϕ) calculated are representative of the actual dust cloud (Equation (1)). l is the distance from the camera to the dust cloud.
- The dust measurement (μ) is representative of the actual dust cloud when the vehicle is in the vicinity of the dust monitor.
- The field of view (FoV) of the video and images was selected so that the road segment is visible at least 20 m but no more than 200 m.
- The dust monitor and the camera were located approximately 10–15 m ( $x_{cd}$ ) apart horizontally from each other.

In this study, we use our URDE dataset [15], which was produced by field experiments conducted on unsealed roads in Victoria, Australia. In the repository, there are two subsets of data, dataset _100 and dataset _897 , which were truncated from the original dataset of approximately 7000 images. Images were extracted from video sequences which were recorded at 30 frames per second.

Let V = $(V_{i}{)}_{i=0}^{n}$ be a labeled set with *n* number of samples, where each sample $V_{i}$ is a frame in a video with $V_{i}\in R^{H\times W\times C}$ . Each $V_{i}$ is of height (*H*) = 1080, width (*W*) = 1920 and channel (*C*) = 3. Let $V_{0}$ be the first frame. Let the *r*th pixel of $V_{i}$ is $p_{r}$ where r∈(h∈H,w∈W) . And $p_{d,r}$ is the probability of dust in pixel $p_{r}$ .

Semantic segmentation of dust from video frames was undertaken from previously trained machine-learning models from our previous works [26], and the three best-performing semantic segmentation machine-learning models were considered for the works presented in this paper. These three models are DeepLabV3_MobileNet_V3 _Large, FCN_Resnet101, and Lraspp_MobileNet_V3_Large. The selected models were trained for URDE dataset_897. Dust-segmented masks were then imposed onto each video frame. The accumulated probabilities of dust in pixels were recorded for each frame, and the average was obtained for 30 frames. Each original image has a corresponding manually annotated image called the ground truth. Original images and corresponding ground truths were used to train the machine learning models. From the processed video, the percentage dust pixel ratio ( ψ ) was calculated according to (Equation (2)).

(1) $$\begin{array}{cc} \mathrm{Average}\;\mathrm{accumulated}\;\mathrm{dust}\;\mathrm{probabilities}\;(\phi ) & =\frac{\sum p_{d,i}}{N} \end{array}$$

(2) $$\begin{array}{cc} \mathrm{Dust}\;\mathrm{pixel}\;\mathrm{ratio}\;(\psi ) & =\frac{1}{30}\sum_{j=0}^{30}\left(\phi \right) \end{array}$$

where $p_{d,i}$ is the probability of dust in the $p_{i}$ pixel, and *N* is the total number of pixels of a video frame. The first Equation (1) calculates static frame dist pixel ratio from each pixel dust probability obtained from semantic segmentation prediction, and the second Equation (2) averages over 30 frames (approx 1 s) to reduce uncertainty noise.

## 3. Experiments

### 3.1. Instrumentation

#### 3.1.1. Dust Monitor

A research-grade real-time dust monitor, the DustTrak™ DRX Aerosol Monitor 8533 from TSI, was used in this study. Using the light-scattering laser photometric method, it can simultaneously measure mass and size fractions continuously every second.

#### 3.1.2. Camera

**Figure 2:**
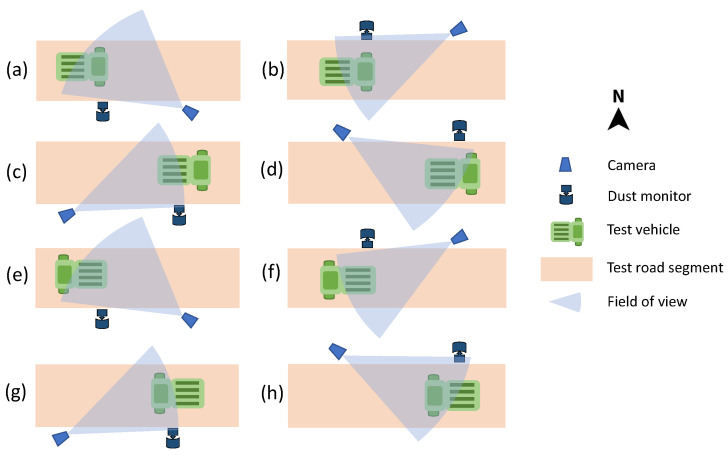
Test setups. Eight different test configurations were used on the same road segment to cover both travel directions and two fields of view per direction. These configurations are as follows: (**a**) Vehicle traveling east with a northwest field of view, (**b**) Vehicle traveling east with a southwest field of view, (**c**) Vehicle traveling east with a northeast field of view, (**d**) Vehicle traveling east with a southeast field of view, (**e**) Vehicle traveling west with a northwest field of view, (**f**) Vehicle traveling west with a southwest field of view, (**g**) Vehicle traveling west with a northeast field of view, (**h**) Vehicle traveling west with a southeast field of view.

The images were collected from videos captured by a Canon EOS 200D direct single-lens reflex (DSLR) camera mounted on a tripod at a fixed focal length and 30 frames per second (fps). The camera was positioned according to the test set-up shown in Figure 2.

#### 3.1.3. Test Vehicle

The test vehicle used for the experiments was a 2002 Toyota Corolla Sedan with an overall height of 1470 mm, an overall length of 4365 mm, an overall width of 1695 mm, a ground clearance (unladen) of 160 mm, a wheelbase of 2600 mm, a kerb weight of 1050 kg, and gross trailer weight brake of 1300 kg.

### 3.2. Test Setups

The test vehicle was driven at different unsealed road segments to produce a dust cloud. The dust cloud was captured from multiple points of view for both travel directions, as shown in Figure 2. The dust monitor was placed in the wind direction so that the dust monitor’s inlet sampled a representative portion of the dust cloud.

## 4. Methods

**Figure 3:**
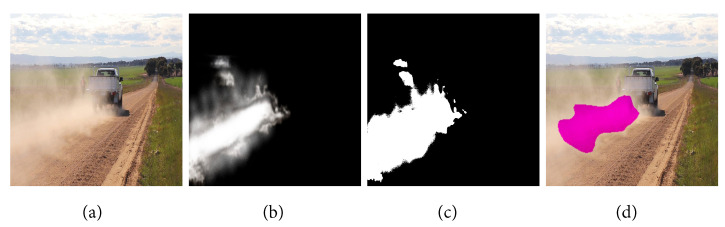
Comparative analysis of dust cloud segmentation methods. (**a**) Original high-resolution field image, (**b**) manually annotated grayscale mask, (**c**) binary segmentation mask derived from the grayscale mask using Otsu’s thresholding method, (**d**) predicted dust segmentation by the machine learning model Lraspp_MobileNet_V3_Large.

**Figure 4:**
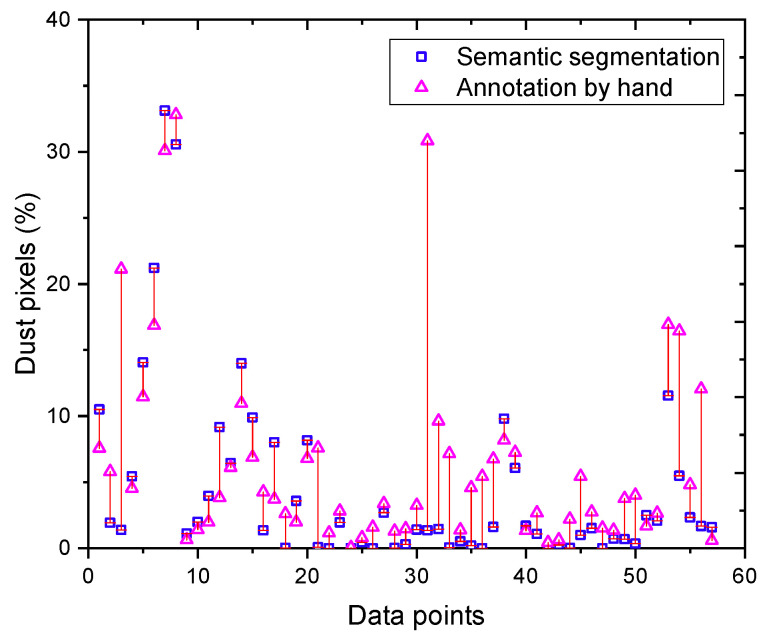
Comparison of dust pixel percentage: machine learning vs. manual annotation.

In order to develop a relationship between features extracted from the images, i.e., the percentage of dust pixels and the actual dust concentrations, first, we investigated the relationship between dust cloud annotation performed by hand and the prediction by an ML model. As shown in Figure 3, some differences exist between the hand-annotated image and the prediction. This is further illustrated in Figure 4 for the dataset. Figure 4 shows differences between machine learning (ML) predictions and manual (hand) annotations of dust pixels. These differences can be due to human error, as people might not always agree on what counts as a dust pixel, model limits as our ML model may miss some nuances in dust patterns, leading to less accurate predictions, limited diversity in data, differences in image resolution between hand annotations, and measurement mistakes. Therefore, it was prudent to develop two relationships for actual dust measurements: one with hand-annotated images and another with ML predictions. The first step encompasses an analysis of original high-resolution field images that capture diverse dust intensities on road surfaces. Accompanying these images are manually annotated grayscale masks, where each pixel’s intensity is indicative of the quantity of dust present. In these masks, darker shades signify minimal or no dust presence (intensity value = 0), while progressively lighter shades up to white represent higher dust concentrations (intensity value = 1). The intermediate shades provide a gradient scale of dust intensity, informing varying dust levels. Additionally, binary segmentation masks are derived from the grayscale masks using Otsu’s thresholding method. This binary format, which comprises only two pixel values (0 or 1), serves as a critical prerequisite for the training of our machine learning model. It simplifies the dust identification process by categorizing the segments into distinct classes. The effectiveness of this approach is further demonstrated in the predicted dust segmentation by the machine learning model Lraspp_MobileNet_V3_Large. This model showcases a capability in identifying dust-laden areas from the surrounding environment [16].

The predictions from segmentation are validated by comparing them with respective field measurements, and air quality thresholds have been imposed on the predictions. Australia has established dust exposure limits of 10 mg/m^3^ for workplaces, so that workers’ breathing zone should not cause adverse health effects or cause undue discomfort [27]. The Australian Standard for PM_10_ is 50 µg/m^3^, measured over a midnight-to-midnight 24 h period. However, most states adopt their own, sometimes stricter, PM_10_ limits. There is currently no national standard for the average one-hour PM_10_. For one-hour PM_10_, Victoria uses the value 80 µg/m^3^ to trigger a ‘poor’ air quality category [28]. The disease control and prevention centers in the United States have set the occupational exposure limit for total dust in flavoring-related work as 15 mg/m^3^ over an 8-h time-weighted average. Multiple dust threshold levels have been established in various countries and jurisdictions. For our study, we opted to adhere to the guidelines set by OSHA [29]. OSHA’s legal airborne permissible exposure limit (PEL) is 15 mg/m^3^ for total dust and 5 mg/m^3^ for respirable dust, averaged over an 8-h work shift.

Correlation 1 was developed using the percentage of dust pixels obtained from manually annotated images. Correlation 2 was developed using the percentage of dust pixels obtained from images segmented with different ML models. The developed correlations are valid for images and videos obtained when the camera captures more than 20 m of the road and less than 100 m from its FoV and the total measured particulate matter is less than 30 mg/m^3^. These are reasonable limitations, because if the dust measurements exceed 30 mg/m^3^ for a prolonged period of time, then the road segment obviously needs to address the dust emissions. If the image obtained captured the dust cloud too close, then it would be an error in selecting the control volume.

### 4.1. Correlation 1

To match a dust cloud with an in situ dust measurement, the generated dust cloud needs to be captured when it is sampled by the dust monitor. A reference object should be in the captured video sequence or the image to make the comparison possible. To effectively solve this problem, we selected a dust monitor as the reference object and designed our field experiments in a way so that the distance between the dust monitor and the camera was the same. To keep the experiments consistent, reproducible, and extendable, the depth data were obtained with respect to a reference image taken beforehand.

Image statistics of the reference object, i.e., the dust monitor, were studied in different crops or angles within multiple images prior to extracting features from the image for the correlation. As the annotation of the images was performed by qualified people, the reference object detection was translation-invariant, viewpoint-invariant, size-invariant and illumination-invariant [30]. There are multiple ways to compare the similarity of an object in different images. An image-based, direct similarity metric could be used, such as the sum of absolute distances, the sum of squared distances [31] or normalized cross correlation [32]. These approaches generalize to template matching, where a convolutional procedure is conducted. For simplicity, in this paper, we use the pixel density resultant ratio to determine similarity, as described below.

The pixel density resultant of the reference image was calculated according to Equation (3). The same for all other images was calculated according to Equation (4).

(3) $$R_{ref}=\sqrt{{\left[\frac{{\left(p_{x}\right)}_{ref}}{{\left(l_{x}\right)}_{ref}}\right]}^{2}+{\left[\frac{{\left(p_{z}\right)}_{ref}}{{\left(l_{z}\right)}_{ref}}\right]}^{2}}$$

where ${\left[p_{x}\right]}_{ref}$ , ${\left[l_{x}\right]}_{ref}$ , ${\left[p_{z}\right]}_{ref}$ , and ${\left[l_{z}\right]}_{ref}$ are the number of pixels along the horizontal length of the dust monitor in the reference image, the horizontal length of the dust monitor in the reference image, the number of pixels along the vertical length of the dust monitor in the reference image, and the vertical length of the dust monitor in the reference image, respectively.

(4) $$R_{i}=\sqrt{{\left[\frac{{\left(p_{x}\right)}_{i}}{{\left(l_{x}\right)}_{i}}\right]}^{2}+{\left[\frac{{\left(p_{z}\right)}_{i}}{{\left(l_{z}\right)}_{i}}\right]}^{2}}$$

where ${\left[p_{x}\right]}_{i}$ , ${\left[l_{x}\right]}_{i}$ , ${\left[p_{z}\right]}_{i}$ and ${\left[l_{z}\right]}_{i}$ are the number of pixels along the horizontal length of the dust monitor in the image of interest, the horizontal length of the dust monitor in the same image, the vertical length of the dust monitor in the image of interest, and the vertical length of the dust monitor in the same image, respectively.

To keep the correlation independent of semantic segmentation, the correlation was developed using ground truths. Each dust pixel percentage obtained using ground truths ( ϕ ) is modified using the ratio $\frac{R_{i}}{R_{ref}}$ , as shown in Equation (5).

(5) $$\phi_{mod}=\phi \times \frac{R_{i}}{R_{ref}}$$

**Figure 5:**
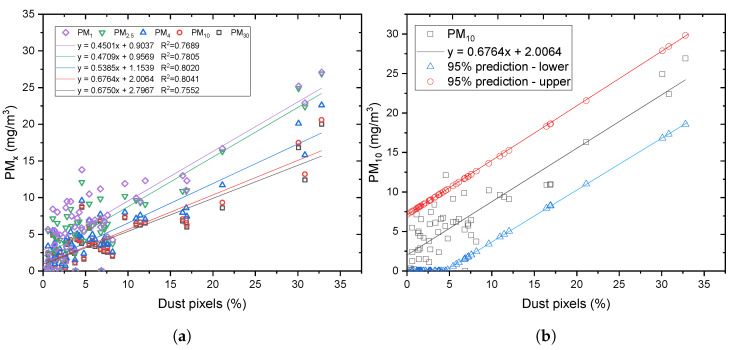
(**a**) Linear correlation between PM_30_, PM_10_, PM_4_, PM_2.5_, and PM_1_, and their respective percentage dust pixels from segmentation. (**b**) Linear correlation between PM_10_ and percentage dust pixels from segmentation together with 95% prediction limits.

Actual field dust measurements were then correlated with $\phi_{mod}$ , as shown in Figure 5.

### 4.2. Correlation 2

**Figure 6:**
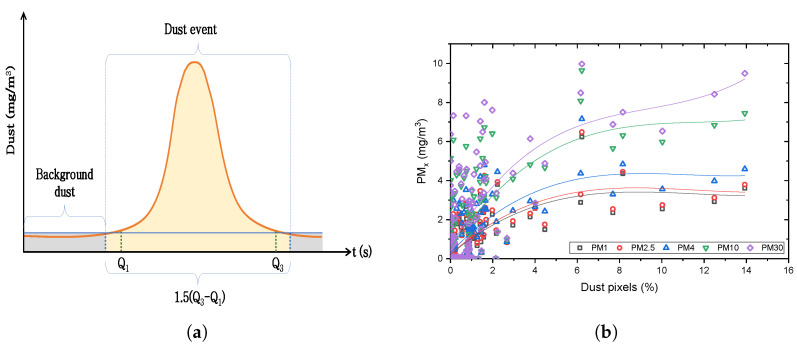
(**a**) Generalized dust event, (**b**) Correlation 2.

Correlation 2 (Table 1) was developed using the segmented results from different video sequences. The dust monitor was turned on in the field experiments before the vehicle started traveling to capture the background dust concentration. Therefore, each experiment has a much higher number of data points for background concentration than the data points for the actual dust event. If all the data points from an experiment were included in developing a correlation, it would be biased and inaccurate. To avoid that, dust events were generalized based on measurements obtained from the dust monitor, as shown in Figure 6a. It is evident that the dust even comprises “outlier” dust readings; therefore, we identified and isolated the dust event by identifying the origin and conclusion of a particular experiment through outlier sampling. The method used was similar to the rule of 1.5(IQR), where IQR is the interquartile range [33]. The first quartile ( $Q_{1}$ ) and third quartile ( $Q_{3}$ ) were calculated, and the IQR was obtained. However, instead of sampling outliers from the interquartile range from each quartile, we used the interquartile range directly. This approach was selected to ensure that adequate portions of the background dust and the dispersion were captured in the event. After dust events had been isolated for all experiments, the correlation was developed as shown in Figure 6b.

### 4.3. Graphical User Interface (GUI)

**Figure 7:**
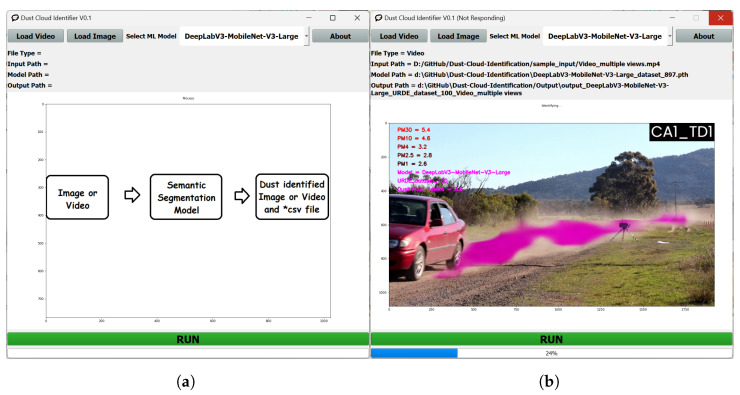
(**a**) GUI home page. (**b**) GUI when it is processing a video sequence.

A graphical user interface (GUI) was developed to dynamically process video or image data and produce the above-discussed analysis on dust clouds. The code and software for this analytical tool are available at https://github.com/RajithaRanasinghe/Dust-Cloud-Identification (accessed on 14 January 2024). A snapshot of the GUI is shown in Figure 7a. A snapshot of the GUI when a video is being processed is shown in Figure 7b. All training, testing and evaluation was performed in a Ryzen 9 5900HX CPU, 32GB RAM, and an NVIDIA RTX3080 Mobile 16GB GPU test environment. Similar platform specifications will be needed to run the executable provided in the above link.

## 5. Results and Validation

**Figure 8:**
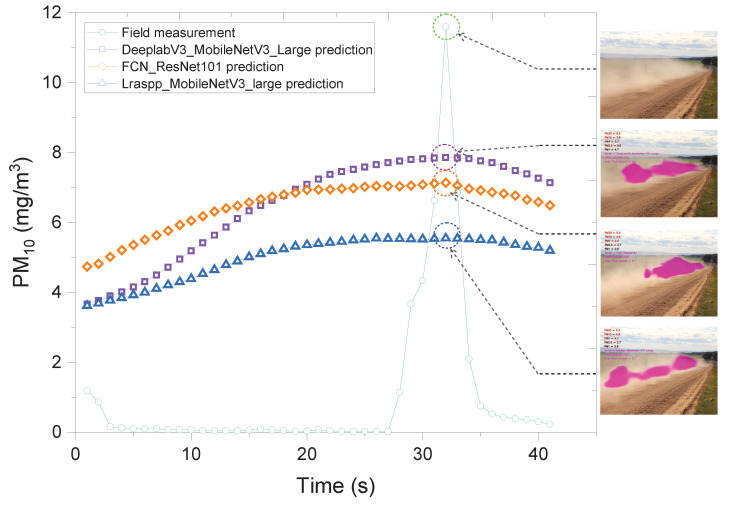
Validation. Field measurement and PM_10_ prediction by the three ML models (correlation 2): DeeplabV3_MobileNetV3_Large, FCN_ResNet101, and Lraspp_MobileNetV3_Large, for video sequence 1.

The developed correlation was validated for an independent set of video sequences. An example of dust predictions for a video segment obtained from all three ML models with corresponding field dust measurements is shown in Figure 8. It is evident that all three ML outcomes are lower than the peak of the actual dust measurement suggesting the ML outcomes underestimate the maximum dust concentration. However, all three ML models overestimate the background dust concentration.

The accuracy of the developed correlations was evaluated by sensitivity (true positive) and specificity (true negative) using the threshold dust concentration $\left(\mu_{d}\right)$ 15 mg/m^3^. The true positives (TP) represent the dust predictions lower than $\left(\mu_{d}\right)$ and are actually lower than $\left(\mu_{d}\right)$ and depicted in quadrant 1. Conversely, the true negatives (TN) are the dust concentrations outside $\left(\mu_{d}\right)$ both in the segmentation predictions and the actual ground concentration and are depicted in quadrant 3. The false positives (FP) represent the dust concentrations that exceed $\left(\mu_{d}\right)$ but are predicted as less than $\left(\mu_{d}\right)$ and are depicted in quadrant 2. The false negatives (FN) are where actual dust predictions suggest that $\left(\mu_{d}\right)$ has been exceeded; however, the actual dust concentrations are less than $\left(\mu_{d}\right)$ and are shown in quadrant 4.

**Figure 9:**
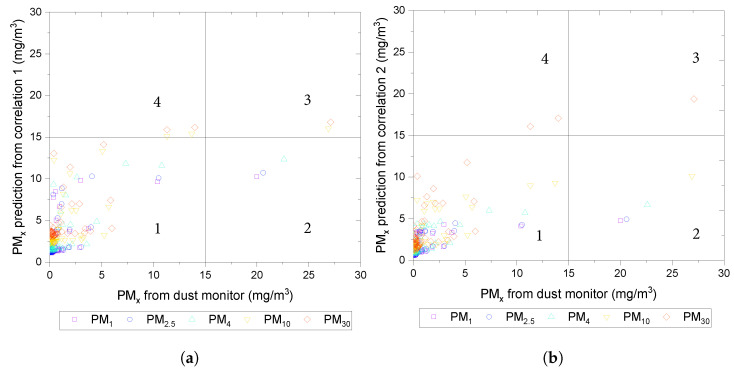
(**a**) Predicted dust concentration vs. actual dust measurement from dust monitor for correlation 1 for PM_30_, PM_10_, PM_4_, PM_2.5_, and PM_1_. (**b**) Predicted dust concentration vs. actual dust measurement from dust monitor for correlation 2 for PM_30_, PM_10_, PM_4_, PM_2.5_, and PM_1_. x=1, 2.5, 4, 10, and 30.

As shown in Figure 9, more than 90% of the data from both correlations fall in quadrant 1, meaning when the dust concentrations are less than the threshold, the segmentation can make an accurate prediction. If a stricter dust threshold is considered, for example, $\mu_{d}$ = 5 mg/m^3^, most of the data in both correlations would still fall in quadrant 1. The number of data points in quadrant 3 would increase in both correlations, suggesting that the correlations can adequately predict dust concentrations in both true positive and true negative cases. In the case of $\mu_{d}$ = 5 mg/m^3^, the rise in false negatives can be observed. This is not non-desirable as a slight overestimation of materials affecting air quality could be preferred among shrewd practitioners.

For both cases of $\mu_{d}$ , the number of data points in quadrant 2 does not change significantly, suggesting that although receiving a false positive is possible, it is negligible.

## 6. Conclusions

This study provides a novel approach to quantifying traffic-induced dust in unsealed roads using semantic segmentation. The proposed method was validated by data collected from multiple field studies, where dust predictions from semantic segmentation were validated using actual in situ dust measurements. For established dust tolerance levels, i.e., 5 and 15 mg/m^3^, it was shown that the proposed method with reasonable accuracy predict traffic-induced dust. This study compares predictions from multiple different ML algorithms as a single algorithm may not make the perfect prediction for a given data set due to its limitations. The main highlights of this paper are summarized as follows.

1. The actual dust measurements from field experiments and dust concentrations predicted by the semantic segmentation demonstrate good correlations subjected to constraints established for field experiments. However, the proposed method is repeatable for different environments and testing equipment, as the main principal hypothesis is validated in this study.
2. Using the user interface we developed, one can go to a road segment of interest where dust emissions need to be assessed and record traffic-induced dust as a video sequence and determine if it exceeds relevant dust thresholds.
3. The developed method to quantify dust from images and videos can be used to assess dust severity with respect to dust exposure limits, thus, can be implemented as a real-world application.

Advanced semantic segmentation algorithms, namely DeepLabV3_MobileNetV3_Large, FCN_ResNet101, and Lraspp_MobileNetV3_large, were employed in conjunction with Otsu’s thresholding method for the identification of dust-laden pixels in this study. This choice of algorithms and thresholding method, while not explicitly accounting for the variability in transmittance across the dust cloud, is aligned with our objective of estimating, rather than precisely quantifying, dust concentrations. The potential over or under-estimation of dust resulting from this approach is deemed acceptable within the parameters of an estimation-focused study. The robustness of our methodology was validated through a comparison with ground truths and actual field dust measurements. This validation process involved capturing images and corresponding dust events in the field alongside real-time dust measurements.

To quantify the correlation between the segmented dust in images and actual in situ measurements, the coefficient of determination (R^2^) was used, further strengthened by applying a 95% confidence interval. This statistical approach underscores the high degree of correlation between our image-based dust estimation and actual field measurements, thereby reinforcing the validity of our method. When compared to existing dust quantification methods, the image segmentation-based approach is recognized as a more cost-effective alternative. Traditional methods typically depend on either less accurate empirical models or expensive dust monitors, whereas the method presented in this study offers a viable solution for dust monitoring, particularly beneficial for applications in environmental monitoring, industrial settings, and public health studies. Future enhancements to the methodology are anticipated, especially in terms of incorporating variability in dust cloud transmittance. These improvements aim to refine the accuracy of dust concentration estimations, reducing any overestimation biases. Furthermore, the authors’ future work will be focused on the automation of scene understanding in unsealed roads in terms of road dust, soil type and traffic, making the approach wholesome.

## 7. Future Works

In this study, we have implemented and tested conventional machine learning models tailored for static image segmentation for dust identification, with each model trained on individual images. While this approach has yielded valuable insights, we anticipate significant enhancements by incorporating cutting-edge machine-learning frameworks capable of video-based analysis. Particularly, architectures like Convolutional Long Short-Term Memory (LSTM) networks, which excel in handling sequential data, and 3D Convolutional Neural Networks (3D CNNs), which extend the capabilities of 2D convolutions by adding time as a third dimension, could be leveraged. These models are adept at capturing the spatial-temporal dynamics in video sequences, making them well-suited for analyzing phenomena such as dust clouds exhibiting spatial and temporal variability. Further exploration into architectures such as Two-Stream Convolutional Networks, which combine spatial and temporal network streams for action recognition in videos, and the more recent Inflated 3D ConvNets (I3D), which inflate filters and pooling kernels into 3D, could provide even more sophisticated tools for dust cloud characterization.

## Figures and Tables

**Table 1:** Correlation 2 regression data.

Equation	$y=a_{0}+a_{1}x+a_{2}x^{2}+a_{3}x^{3}$				
Plot	PM_1_	PM_2.5_	PM_4_	PM_10_	PM_30_
$a_{0}$	0.207 ± 0.053	0.231 ± 0.056	0.304 ± 0.067	0.543 ± 0.109	0.612 ± 0.126
$a_{1}$	0.913 ± 0.110	0.970 ± 0.116	1.152 ± 0.138	1.594 ± 0.225	1.868 ± 0.260
$a_{2}$	−0.084 ± 0.028	−0.089 ± 0.029	−0.106 ± 0.035	−0.134 ± 0.056	−0.179 ± 0.065
$a_{3}$	0.002 ± 0.002	0.003 ± 0.002	0.003 ± 0.003	0.004 ± 0.003	0.006 ± 0.004
Residual sum of squares	89.10	98.72	139.15	370.99	495.99
$R^{2}$	0.55	0.55	0.55	0.51	0.51
Adjusted $R^{2}$	0.54	0.54	0.55	0.50	0.50

## Data Availability

The URDE dataset is publicly available at https://doi.org/10.6084/m9.figshare.20459784.v3 (accessed on 15 November 2022).

## Abbreviations

The following abbreviations are used in this manuscript:

MDPI	Multidisciplinary Digital Publishing Institute
PM	Particulate Matter
USEPA	United States Environmental Protection Agency
ML	Machine Learning
FCN	Fully Convolutional Network
LR-ASPP	Lite Reduced Atrous Spatial Pyramid Pooling
DSC	Dice Similarity Coefficient
FoV	Field of View
URDE	Unsealed Road Dust Dataset
DSLR	Direct Single Lens Reflex
fps	frames per second
GUI	Graphical User Interface
GPU	Graphics Processing Unit
CPU	Central Processing Unit
